# The role of contraception in preventing HIV-positive births: global estimates and projections

**DOI:** 10.1186/s12889-021-10570-w

**Published:** 2021-03-19

**Authors:** Jennifer Sherwood, Elise Lankiewicz, Beirne Roose-Snyder, Bergen Cooper, Austin Jones, Brian Honermann

**Affiliations:** 1 Public Policy Office, amfAR, Foundation for AIDS Research, 1100 Vermont Avenue NW, Suite 600, District of Columbia, Washington, DC, 20005 USA; 2 Department of Population, Family, and Reproductive Health, Johns Hopkins Bloomberg School of Public Health, Baltimore, MD USA; 3 Center for Health and Gender Equity (CHANGE), Washington DC, USA

**Keywords:** Contraception, HIV, Women, Antenatal HIV, Prevention, Family planning

## Abstract

**Background:**

Meeting the contraceptive needs of women living with HIV (WLHIV) has primary health benefits for women, in addition to being a key element to prevent mother-to-child HIV transmission. This analysis will estimate the current number of infant HIV infections prevented by contraception in the era of increased HIV treatment coverage and; 2) model the additional HIV benefits of preventing unintended births to WLHIV.

**Methods:**

Secondary data analysis was conducted using publicly available data from the United Nations Programme on HIV/AIDS (UNAIDS) and Population Division, Demographic Health Surveys, and peer-review literature. National data from 70 countries, that had a UNAIDS estimate for the number of WLHIV nationally, were combined into country-level models. Models estimated the current number of infant HIV infections averted by contraception annually and potentially averted if unintended births to WLHIV were prevented. Estimates take into account pregnancy and live birth rates, contraceptive coverage, contraceptive method mix and failure rates, and HIV treatment coverage during pregnancy to prevent mother to child transmission.

**Results:**

Contraception use among WLHIV prevents an estimated 43,559 new infant HIV infections annually across 70 countries. Countries with the largest number of infant infections averted by contraception included South Africa (9441), Nigeria (4195), Kenya (3508), Zimbabwe (2586), and India (2145). Preventing unintended births to WLHIV could avert an additional 43,768 new infant infections per year, with the greatest potential gains to be made in South Africa (12,036), Nigeria (2770), Uganda (2552), and the Democratic Republic of the Congo (2324).

**Conclusions:**

Contraception continues to play an integral role in global HIV prevention efforts in the era of increasing HIV treatment coverage, especially in sub-Saharan Africa. Broad contraceptive availability, increased contraceptive voluntarism and method mix are key components to preventing unintended births and ending new infant HIV infections worldwide.

**Supplementary Information:**

The online version contains supplementary material available at 10.1186/s12889-021-10570-w.

## Background

There are an estimated 14.7 million women of reproductive age (15–49) living with HIV, accounting for nearly 40% of the global HIV burden. In East and Southern Africa, the regions most impacted by HIV, adult females (15+) make up the majority (58%) of people living with HIV [1]. Accordingly, addressing the impact of HIV on this population is central to efforts to end the HIV pandemic and improve the wellbeing of millions of women across the globe; moreover, better serving this population may further HIV prevention efforts given the potential of mother-to-child transmission of the virus during pregnancy, labor or breastfeeding. In 2018, there were an estimated 160,000 new HIV cases among children aged 0–9, the overwhelming majority of which are attributable to MTCT [2].

Efforts to prevent mother-to-child transmission (PMTCT) have been focused in four areas: primary prevention of HIV among women of childbearing age, preventing unintended pregnancies among women living with HIV (WLHIV), preventing transmission between WLHIV and their infants, and providing treatment care and support to mothers living with HIV, their children, and families [3]. Accordingly, two key components of these efforts have been ensuring pregnant women are on effective antiretroviral therapy (ART) and providing accessible voluntary contraceptive resources to prevent unintended pregnancies. Continual ART for mothers and short-term regimens for newborns can reduce the likelihood of HIV transmission from 15 to 45% to less than 5% [4]. Globally, ART coverage of women during pregnancy has nearly doubled since 2010, from 44 to 82%; however, wide country and regional disparities remain [1].

In contrast to the recent progress made in ART coverage for pregnant women, growth in contraceptive prevalence has followed a slower trajectory, with persistent demonstration of unmet need globally [5]. In 2017, an estimated 214 million women in low and middle income countries wished to avoid pregnancy but were not using a modern contraceptive method [6]. The highest proportion of these women were in Sub-Saharan Africa, the region which also bears the highest burden of HIV [1]. Even where modern contraception is available, women and girls may face limited options in method mix. Limited contraceptive options represents an additional barrier in addressing unintended pregnancies, given that expanded options are associated with increased contraceptive prevalence [7].

Globally, the proportion of births that are unintended is also substantial. From the period of 2010–2014, nearly a quarter of births globally were unintended, and in Southern Africa, the Caribbean, and South America, more than half of births were unintended [8]. Unintended births are commonly defined as births that occurred sooner in one’s life than desired or were not wanted at all [9]. Given that the primary contributors to unintended births include non-use or unmet need for family planning services and contraceptive failure [10], it follows that a critical avenue to ensuring women’s full reproductive control includes increasing access to voluntary family planning services.

Voluntarism in family planning services refers to the ability of patients to make a full, free and informed choice about their reproduction and reproductive health services, without any force, coercion or undue incentivization [11]. Voluntarism is vital in all health services, but the need to safeguard voluntarism and contraceptive choice for WLHIV is particularly acute given persistent reports of forced or coercive experiences with family planning programs [12, 13]. Throughout this paper, any reference or recommendations related to family planning programs or expansion of contraceptive access are referring to services implemented with full informed choice and rigorous human rights safeguards in line with World Health Organizations guidelines [14].

Meeting women’s contraceptive needs has numerous health benefits for the individual, which deserve discussion, including reducing maternal mortality and morbidity [15, 16]. However, the primary focus of this paper is on the additional HIV benefits of meeting the contraceptive needs of WLHIV – a key element of PMTCT efforts, which has yet to be fully realized [17]. Previous estimates (2008) have calculated that contraception prevented over 200,000 new HIV positive births annually in President’s Emergency Plan for AIDS Relief (PEPFAR) countries (*n* = 15) [18]. This previous estimate, however, assumed the absence of ART coverage for PMTCT. Accounting for recent positive changes in ART coverage for PMTCT and contraceptive method mix (CMM), the current analysis builds on previous work to examine 1) The number of new HIV cases averted annually due to contraceptive usage, and 2) the number of additional HIV cases that may be averted if unintended births to WLHIV were prevented.

## Methods

### Data sources

All data are sourced from publicly available datasets from the Joint United Nations Programme on HIV and AIDS (UNAIDS) [19], the United Nations Population Division, Department of Economic and Social Affairs (UN DESA) [20], country Demographic Health Surveys [21], Family Planning 2020 [22], Guttmacher [23], and other peer-reviewed literature. The full set of data sources is available in Table 1. All countries with an UNAIDS estimate for the national number of WLHIV were included, except countries for which the total number of WLHIV (15–49) was < 10,000, given their negligible contribution to the final outcome of interest.

**Table 1:** Data sources

Indicator	Description	Main Source(s)	Notes
WLHIV of reproductive age	Number of WLHIV aged 15 to 49 years old, country specific data	Joint United Nations Programme on HIV and AIDS (UNAIDS), 2018 [1]	Global Health Data Exchange (GHDx) 2018 data were used where UNAIDS data were not available for 2018 [24]
Contraception prevalence rate (CPR)	Proportion of married or in-union women using any modern method of contraception, country specific data	UN, Department of Economic and Social Affairs, Population Division, 2019 [25]	Most recent survey year data available was used
Annual pregnancy rate (PR)	Pregnancy rate among women among women aged 15–44, sub-regional data	Sedgh et al., 2016 [26]	Pregnancies include all live births, abortions, and miscarriages
Contraceptive method mix (CMM)	The proportion of total family planning users using each modern method of contraception, country specific data	Family Planning 2020 (FP2020), 2019 [22]	Modern methods include sterilization (female), sterilization (male), IUD, implant, injectable, pill, male condoms, and Lactational Amenorrhea Method (LAM)
Contraceptive method-specific failure rates	Median failure rate in first 12 months of use by method	Guttmacher Institute, 2016 [23]	Uses DHS survey data from 43 countries, 1990–2013
ART coverage for PMTCT	Proportion of pregnant WLHIV who receive ART for PMTCT, country specific data	UNAIDS, 2018 [1]	Where 2018 UNAIDS data were not available, UNAIDS reports using 2017 and 2016 data were used [27–29]. Other sources include: the Centers for Disease Control and Prevention (CDC), USA [30]; Public Health England, United Kingdom [31]; Clark, 2017, Russia [32]. Countries with data still missing were set at the UNAIDS global average (0.82)
Annual birth rate 15–49	Annual rate of live births to women 15–49 by country & sub-region	United Nations Population Division [20]	Used age-specific fertility rates and female population by age to create weighted annual births among women 15–49
Proportion of births unintended	Proportion of births that arise from unintended pregnancies, country-level data	DHS Stat Compiler [21]	For countries where DHS data were not available or where DHS data pre-dated 2000, the corresponding sub-regional estimate from Bearak et al., 2018 [8] was used

### Data analysis objective 1: calculating total new infant infections currently averted by contraception

First, births averted by current contraception use among WLHIV were calculated as follows; where WLHIV is the number of WLHIV of reproductive age (15–49) [1], CPR is the national contraception prevalence rate [25], CFR is the country-specific contraception failure rate, PR is the annual pregnancy rate [26], and BR is the estimated live birth rate [20]:

$$ Births\ averted\  by\  contraception\  use\  among\ WLHIV=\left( WLHIV\ast CPR\ast PR-\left( CFR\ast WLHIV\ast CPR\right)\right)\ast \left(\frac{BR}{PR}\right) $$

In this calculation, estimated CPR is not specific to WLHIV, instead taking into account the use any modern contraception method among married or in-union women [25]. Country-specific contraceptive failure rates (CFR) were calculated using the proportion of women using each modern contraceptive method [22] and the contraceptive method-specific failure rates [23] to calculate the average CFR for each country based on method mix. Countries without data on the proportion of women using each contraceptive method were assigned a CFR of 0.031. The represents a simple average of country CFRs with available data. Overall, country CFR ranged from 0.015 to 0.052. Countries assigned the global CFR average (*n* = 25) are Argentina, Botswana, Brazil, China, Columbia, Dominican Republic, Ecuador, Equatorial Guinea, Eswatini, France, Gabon, Guatemala, Iran, Italy, Malaysia, Mexico, Namibia, Peru, Russian Federation, Spain, Thailand, Ukraine, United Kingdom, United States, and Venezuela.

Pregnancy and birth rates were calculated at the sub-regional level – referring to the division of continents into smaller geographic country groupings (e.g., Asia is split into Eastern, South-central, Southeastern, and Central regions). Pregnancy and birth rates were not universially available at the country-level. To adjust for the proportion of pregnancies which would not ultimately result in a live birth (due to spontaneous or induced abortion), sub-regional birth rates are divided by sub-regional pregnancy rates.

Next, the calculated number of births averted by contraception use among WLHIV in each country was used to estimate the number of new infant HIV infections that are currently being prevented by contraception. These calculations were completed in two parts: 1) Among births to WLHIV who were on ART during pregnancy, assuming 4% transmission and; 2) Among births to WLHIV who were not on ART during pregnancy, assuming 30% transmission [4]. ART coverage during pregnancy, labor, and breastfeeding (PMTCT coverage) was recorded from UNAIDS 2018 estimates [33]. Countries without data on PMTCT coverage (France, Italy, and Spain) were assigned the global average of 0.82, and countries with coverage of > 0.95 were assigned a value of 0.95. The number of new infant HIV cases averted from births to women on and not on ART was calculated as follows, where PMTCT is country-level PMTCT coverage and BA_contraception is the number of births averted by contraception to WLHIV:

$$ Total\ infant\  HIV\  cases\ averted\  by\  contraception=\left( PMTCT\ast B{A}_{\_ contraception}\ast 0.04\ \right)+\left(\left(1- PMTCT\right)\ast B{A}_{\_ contraception}\ast 0.30\right) $$

### Data analysis objective 2: calculating additional infant infections averted by preventing unintended births to WLHIV

First, unintended births to WLHIV by country were calculated as follows, where WLHIV is the number of WLHIV of reproductive age (15–49), BR is the country- level live birth rate [20] among women, and pBU is the proportion of births which are estimated to be unintended [21]:

$$ Unintended\ birth\ to\ WLHIV= WLHIV\ast BR\ast pBU $$

Here, both the country-level birth rate and the proportions of births which are estimated to be unintended are calculated for women overall and not specifically for WLHIV. These data are unavailable for WLHIV. The estimated country-level birth rate was found by weighting age-specific fertility rates by age-distributed population estimates for women 15–49 in each country [20].

 Next, the number of unintended births to WLHIV in each country were used to estimate the additional infant HIV infections averted if unintended births to WLHIV were prevented. This was calculated separately for WLHIV currently on ART during pregnancy and not on ART during pregnancy as described above. Calculations were completed as follows, where PMTCT is country-level PMTCT coverage, and UB_WLHIV is the number of unintended births to WLHIV by country.

$$ Total\ additional\ infant\  HIV\  infections\ averted=\left( PMTCT\ast UB\_ WLHIV\ast 0.04\ \right)+\left(\left(1- PMTCT\right)\ast UB\_ WLHIV\ast 0.30\right) $$

## Results

### Contraceptive failure rates

Among the 70 countries in this analysis, the estimated country-level contraception failure rate (CFR) was highest in the Central African Republic (52 per 1000 women using contraception), where the primary modern method used is the pill. This was closely followed by the Democratic Republic of the Congo (49 per 1000), where the primary modern method used is male condoms. CFRs were lowest in Uzbekistan (17 per 1000), where the primary modern method used is the IUD, followed by Ethiopia (18 per 1000), where higher proportions of women are using the implant compared toother African settings. In South Africa (CFR 30 per 1000), 47% of modern contraception users are using an injectable birth control (data not shown see Additional file 1).

### New infant HIV infections currently averted by contraception

**Fig. 1:**
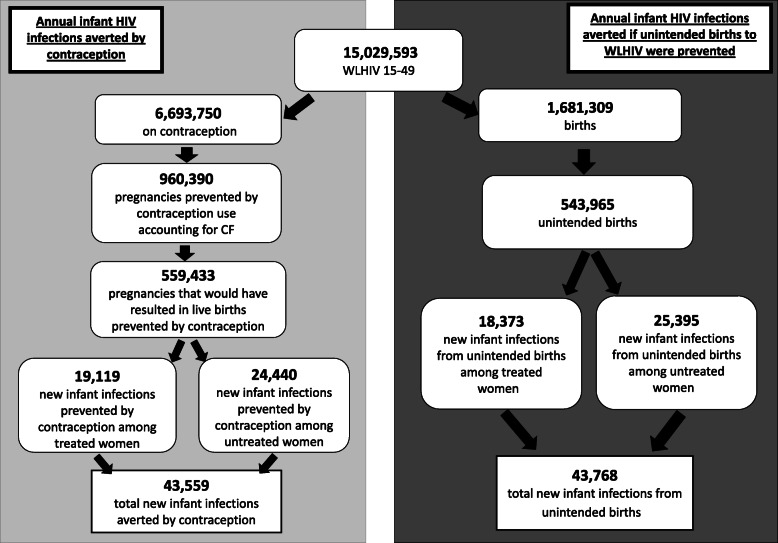
Summary of Annual Model Estimates (70 countries)

Across the 70 included countries, current contraceptive use by WLHIV was estimated to be averting 43,559 new HIV infant infections annually (Fig. 1). Full results available in Additional file 2. Countries with the largest number of infant infections averted by contraception included South Africa (9441), Nigeria (4195), Kenya (3508), Zimbabwe (2586), and India (2145) (Table 2).

**Table 2:** Number of HIV positive births averted by contraception annually, top 30 countries

Country	WLHIV aged 15–49^a^ (A)	WLHIV (15–49) on modern contraception^b^ (B)	Pregnancies to WLHIV averted by contraception^c^ (C)	Births to WLHIV averted by contraception ^d^ (D)	HIV- positive births averted by contraception^f^		
					Treated	Untreated	Total
South Africa	4,050,000	2,187,000	204,530	127,930	4452	4989	9441
Nigeria	860,000	161,680	36,138	22,604	398	3798	4195
Kenya	740,000	434,380	96,648	55,337	2014	1494	3508
Zimbabwe	600,000	394,800	81,224	46,506	1749	837	2586
India	655,544	313,350	26,808	14,898	358	1788	2145
Malawi	480,000	278,880	63,411	36,307	1380	545	1924
Uganda	650,000	235,950	52,899	30,288	1127	636	1763
Indonesia	217,800	128,066	12,919	6709	40	1711	1751
Zambia	580,000	259,260	57,572	32,963	1253	494	1747
Mozambique	1,060,000	268,180	57,384	32,856	1249	493	1741
Tanzania	720,000	230,400	50,511	28,921	1076	607	1683
Ethiopia	324,000	122,472	27,981	16,021	590	384	974
Cameroon	277,000	58,170	13,972	8748	280	525	805
Angola	179,000	22,375	5549	3474	53	646	699
Brazil	244,313	189,831	16,895	8172	278	368	646
Ghana	170,000	42,500	9822	6144	194	387	581
Lesotho	152,000	90,896	7737	4840	149	334	483
DRC	234,000	17,550	4105	2570	45	432	477
Mali	74,000	11,174	2607	1631	16	372	387
Eswatini	103,000	67,465	6274	3924	124	247	371
Côte d’Ivoire	205,000	40,180	8682	5431	195	163	358
Thailand	170,000	128,350	12,322	6399	243	96	339
Rwanda	103,000	48,925	10,823	6197	235	93	328
Congo	46,900	8677	1999	1252	13	282	294
U.S.	226,698	153,475	10,590	5509	209	83	292
Russia	278,415	153,128	12,097	5068	193	76	269
Botswana	159,000	81,408	7571	4736	180	71	251
Madagascar	18,578	7171	1613	924	4	247	251
Myanmar	85,000	43,605	4287	2226	71	134	205
Viet Nam	60,000	39,000	3706	1925	62	110	172

^a^ Calculated as women living with HIV aged 15+ − WLHIV aged 50+

^b^ Calculated as A * Contraceptive prevalence rate

^c^ Calculated as (B * pregnancy rate) – (B * contraception failure rate)

^d^ Calculated as C * proportion of pregnancies resulting in live birth (birth rate/pregnancy rate)

^f^ Calculated as (D * Coverage of pregnant women for preventing mother-to-child transmission [PMTCT])* 0.04 estimated transmission among those on treatment) + (D * (1 – coverage of pregnant women for PMTCT) * 0.30 estimated transmission among those not on treatment)

The majority of new infant cases (24,440/43,559, 56%) currently being averted by contraception were among women not receiving ART during pregnancy (untreated). However, contraception is still preventing a significant number of total new infant HIV cases (19,119 or 44%) among women who were on ART during pregnancy (treated). While the overall ratio of infant HIV cases averted among untreated vs. treated women was about 60:40, there was wide country variability. In some countries, such as Malawi, Zambia, and Mozambique, contraception is preventing over twice as many new infant infections among women on ART than women not on ART (1380 vs. 545; 1253 vs. 494; and 1249 vs. 493 respectively).

### Potential infant HIV infections averted if unintended births among WLHIV were prevented

Our model estimates an additional 43,768 new HIV cases could be averted annually if unintended births among WLHIV were prevented (Fig. 1). Countries with the largest number of potential gains to be made in preventing infant HIV cases are in sub-Saharan Africa including South Africa (12,036), Nigeria (2770), Uganda (2552), the Democratic Republic of the Congo (2324), and Angola (2147) (Table 3). Full results are available in Additional file 3.

**Table 3:** Number of additional infant HIV infections averted if unintended births to WLHIV were prevented, top 30 countries

Country	No of WLHIV aged 15–49^a^ (A)	No of births to WLHIV annually^b^ (B)	No of unintended births to WLHIV annually^c^ (C)	Total new infant infections from unintended births to WLHIV^d^		
				Treated	Untreated	Total
South Africa	4,050,000	302,024	163,093	5676	6361	12,036
Nigeria	860,000	142,123	14,923	263	2507	2770
Uganda	650,000	105,659	43,848	1631	921	2552
DRC	234,000	43,479	12,522	220	2104	2324
Angola	179,000	31,753	10,669	162	1984	2147
Tanzania	720,000	112,564	34,895	1298	733	2031
Kenya	740,000	82,445	29,433	1071	795	1866
Zambia	580,000	86,809	33,335	1267	500	1767
Malawi	480,000	67,712	27,694	1052	415	1468
Mozambique	1,060,000	167,026	24,720	939	371	1310
Zimbabwe	600,000	69,895	22,716	854	409	1263
Cameroon	277,000	40,647	9633	308	578	886
Lesotho	152,000	15,567	8017	247	553	800
South Sudan	87,000	12,901	4773	107	630	737
Ethiopia	324,000	43,585	11,071	407	266	673
Eswatini	103,000	9982	6359	201	401	602
Ghana	170,000	20,201	6242	197	393	590
Indonesia	217,800	14,774	2246	13	573	586
India	655,544	45,359	3946	95	474	568
Brazil	244,313	12,491	6870	234	309	543
Côte d’Ivoire	205,000	30,598	7405	267	222	489
Mali	74,000	13,867	1941	19	443	461
Congo	46,900	6392	1796	18	404	422
Botswana	159,000	14,168	7651	291	115	406
Haiti	73,000	6685	3790	126	193	319
Namibia	87,000	9485	4847	184	73	257
Rwanda	103,000	12,931	4629	176	69	245
U.S	226,698	13,041	4564	173	68	242
CAR	48,000	7353	2059	58	179	238
Equatorial Guinea	26,300	3984	1116	22	167	190

^a^ Calculated as women living with HIV (WLHIV) aged 15+ − WLHIV aged 50+

^b^ Calculated as A * annual birth rate

^c^ Calculated as B * proportion of births that are unintended

^d^ Calculated as (C * coverage of pregnant women for PMTCT * 0.04 estimated transmission among those on treatment) + (C * (1 - coverage of pregnant women for PMTCT) * 0.3 estimated transmission among those on treatment)

If all unintended births to WLHIV were prevented, the largest number of new infant HIV infections averted would be to women not on ART (25,395 / 43,768, or 58%). However, gains would also be made among women on ART, with an additional 18,373 (42%) new infant HIV infections averted among women on ART. Again, there was significant country variability with many countries (25/70, 36%) projected to prevent more infant HIV infections among women on ART than women not on ART if all unintended births were prevented. Of note, countries that have greater than or equal to 95% coverage of ART for pregnant women in our analysis (*n* = 17/70) account for 14% (6098/ 43,768) of the additional infant infections that could be averted if unintended births to WLHIV were prevented.

## Discussion

The results of this analysis show the central role contraception continues to play in preventing new infant HIV infections in the era of increased ART coverage – estimating that contraception is preventing 43,559 infant infections per year in 70 countries. In comparison to previous estimates from 2005 to 2009 [18, 34–36], the current model finds a smaller overall number of infant infections averted by contraception (43,000 vs. 200,000 in PEPFAR countries). This is an unsurprising result given the increase in ART coverage in the past decade. Indeed, since 2010, global ART coverage during pregnancy has increased from 44 to 82% with the largest gains made in Southern and Eastern Africa [1]. However, our results do show similar geographical rankings as previous models, with Southern and Eastern Africa remaining the regions with the greatest potential to prevent infant HIV infections by preventing unintended births.

ART coverage is fundamental for protecting women’s own health and remains a top priority in fighting the HIV pandemic. However, current results show that even in the context of continued scale-up of ART, contraception plays a significant role in the prevention of new infant HIV infections. For example, South Africa has relatively high ART coverage of women during pregnancy (> 87%), which prevents the majority of new infant infections. However, over 12,000 additional infant HIV cases could be averted annually in South Africa if unintended births to WLHIV were prevented with voluntary contraceptive access. Even in countries where ART coverage for PMTCT is excellent (≥95%), unintended births still contribute around 14% of new infant infections. Results demonstrate that improving contraceptive access remains critical to prevent HIV even in these settings with high ART coverage. To meet the health needs of WLHIV and end MTCT, it is critical that both voluntary efforts to expand ART coverage for WLHIV and prevent unintended pregnancies are aggressively implemented.

UNAIDS and PEPFAR established the goal of reducing the annual number of children newly infected with HIV to fewer than 20,000 by 2020 in their “Start Free, Stay Free, AIDS Free” framework [33]. While progress has been made, the world has not yet met this goal. In 2018, 160,000 children were newly diagnosed with HIV and global ART coverage for PMTCT remained at 82% [33]. As work to close this gap intensifies, meeting the voluntary contraceptive needs of WLHIV must remain a central pillar of this effort. One avenue to help meet this goal is to increase integration of ART for PMTCT and family planning programming. Strategies for integration include providing both services in the same location or through active coordinated care referral systems [37]. A number of studies have demonstrated integration of these services resulted in increased contraception uptake, increased use of more effective methods, and reduced pregnancy rates among those at risk for or living with HIV [38–41]. Strong international and national funding for family planning services is also critical. Contraceptive stock outs and shortages are common globally and unmet need for contraception continues to outpace global funding for contraceptive commodities [42]. National governments and donors who are serious about goals to end MTCT will also prioritize meeting women’s contraceptive needs as a key strategy post-2020.

In addition to increasing access to any modern method of contraception, expanding the variety of contraceptive methods available may also play a role in increasing overall contraceptive prevalence [7]. Many countries included in this analysis have a skewed contraceptive method mix, where 50% or more of modern contraception users rely on a single method. Ensuring women have a variety of options to meet reproductive needs and preferences may encourage overall contraception use. Many donors, particularly those with country focuses, have invested in the scale-up of a single contraceptive method, an investment that can inadvertently skew method mix [43]. While commitment to novel and underused methods is commendable, donors and national governments must coordinate to provide method mix of desired contraceptives not at a national, but at a clinical and site level [14]. The thoughtful global consensus language found in FP 2020 commitments [44] still requires more actualizing to make sure that the informed choice of individuals is supported by actual commodity choice at the clinic.

The value of increased CMM is in providing greater choice to users and extends beyond HIV prevention, but could also lower CFR. The modern methods included in the analysis had an estimated range of CFR from 0.06 for the pill to 0.01 for both IUDs and implants [23]. While the more effective contraception methods with lower CFR may not be preferable or appropriate for all women, ensuring access should they want them is a valuable strategy to lower CFR and decrease unwanted births to WLHIV and others. Long-acting reversible contraception (LARC) uptake is on the rise in Sub-Saharan Africa, yet gains have been more significant among wealthier, higher educated, and urban women [45], leaving gaps in availability and accessibility for many groups of women that may also be less likely to have access to effective ART during pregnancy. Addressing barriers related to awareness, supply, and provider training are all of importance in ensuring access to women’s desired form of contraception.

There are several limitations to this analysis. While every effort was made to use consistent and quality data sources across countries, data gaps prompted some differential use of data by country, survey type, or year of data collection. Firstly, country-level data was not available for all indicators. In these cases, sub-regional data were used, which may have masked country-level disparities. Secondly, data on CFR and PMTCT coverage were missing for 25 and 3 countries respectively. In these cases, missing data was imputed with global averages. Mean imputation of these values may have again reduced differences between countries. However, countries with imputed data (*n* = 28) only contribute approximately 2600 events (6%) to the estimated 43,559 new HIV infant infections averted annually by contraceptive use. Relatively minor adjustments to imputed data for these countries would not greatly alter the overall estimation of infant infections adverted. Thirdly, many of the secondary datasets used in this analysis are themselves modelled and include uncertainty bounds. For example, UNAIDS HIV prevalence estimates for women are reported as a range. In these cases, our models used the median point estimates, which may have resulted in over or under estimations of the final outcome of interest.

For some indicators, including annual birth rate, annual pregnancy rate, proportion of births unintended, contraceptive prevalence rate, and CMM, country-level data specific to WLHIV are unavailable. In these cases, data from the overall population of women were substituted. Existing literature suggests there may be differences for these indicators between WLHIV and the general population of women which may affect model estimates. For example, WLHIV may have a higher [46, 47] or lower [48] overall contraceptive prevalence rate compared to the general population. These size of these differences vary by country. For example, in Tanzania, WLHIV were found to have a contraception prevalence of 54% compared to 32% in the general population [46] while among WLHIV in Togo, contraception prevalence was 74.7% compared to 19.9% in the general population [47]. WLHIV may also have more difficulty accessing diverse contraception methods [49], resulting in a different CCM and CFR as compared to a country’s general population. WLHIV also may have a different rate of unintended births [50]. It is difficult to confirm if these differences contributed to an under or overestimation of the outcomes. Finally, this model only includes births to women aged 15–49, however births can occur to women over 50 and under 15 [51], which are not included, but likely contributed to an underestimation of total infant HIV infections averted.

## Conclusion

The analysis shows that contraception continues to play a significant role in the prevention of new infant HIV infections even in the era of increasing global ART coverage. Continued progress towards global goals to end MTCT will rely on the continued scale-up of ART coverage for women as well as ensuring the availability of contraception for women wishing to prevent pregnancy. Increasing CMM to meet the diverse needs of women is a necessity and should be seen as integral to both the effort to end HIV in children and the women’s health movement.

## Supplementary Information

**Additional file 1.**

**Additional file 2.**

**Additional file 3.**

## Data Availability

Pre-downloaded data sets are available from the corresponding author on reasonable request. Datasets used for the estimates of women living with HIV are available from UNAIDS [https://aidsinfo.unaids.org/] [1] and the Global Health Data Exchange [http://ghdx.healthdata.org/gbd-results-tool2017] [24]. The dataset used for contraception prevalence is available from the UNDP [https://www.un.org/en/development/desa/population/publications/dataset/contraception/wcu2019/UNPD_WCU2019_Country_Data_Survey-Based.xlsx] [25]. The dataset for pregnancy rate is available from Sedgh et al. [10.1111/j.1728-4465.2014.00393.x] [26]. The dataset used for contraception method mix is available from Family Planning 2020 [http://progress.familyplanning2020.org/resources2019] [22]. The dataset used for contraception failure rates is available from the Guttmacher Institute [https://www.guttmacher.org/report/contraceptive-failure-rates-in-developing-world] [23]. The dataset used for PMCTCT Coverage is available from UNAIDS [https://aidsinfo.unaids.org/] [1]. Additional PMTCT data were from the CDC [https://www.cdc.gov/hiv/group/gender/pregnantwomen/index.html] [30], Public Health England [https://assets.publishing.service.gov.uk/government/uploads/system/uploads/attachment_data/file/602942/HIV_in_the_UK_report.pdf] [31], and Clark [10.1016/S0140-6736(16)31480-5] [32]. The dataset used for birth rates is available from the UNDP [https://population.un.org/wpp/Download/Standard/Population/] [20]. The dataset used for unintended births is available from the DHS Stat Compiler [http://www.statcompiler.com] [21]. Additional unintended births data is from Bearak et al. [10.1016/S2214-109X(18)30029-9] [8].

## Abbreviations

ART: Antiretroviral therapy
BR: Birth rate
CMM: Contraceptive method mix
CPR: Contraceptive prevalence rate
DHS: Demographic Health Survey
HIV: Human Immunodeficiency Virus
IUD: Intrauterine Device
LARC: Long-acting reversible contraception
MTCT: Mother to child transmission
PEPFAR: President’s Emergency Plan for AIDS Relief
PMTCT: Prevention of mother to child transmission
PR: Pregnancy rate
UNAIDS: United Nations Programme on HIV/AIDS
WLHIV: Women living with HIV
