# Supplementary material for: The role of contraception in preventing HIV-positive births: global estimates and projections
Source: BMC Public Health. 2021 Mar 19;21:536. doi: 10.1186/s12889-021-10570-w (PMC7977320; doi:10.1186/s12889-021-10570-w)
Supplement: Supplementary file 1 — Additional file 1. [file 12889_2021_10570_MOESM1_ESM.pdf]

Additional File 1: Contraceptive failure rate by country for modern users in countries with available data (n=44) ^

Country	Proportion of women using each modern method of contraception					CFR by country for modern users*
	IUD (A)	Implant (B)	Injectable (C)	Pill (D)	Condoms, male (E)	
CAR	0	0.022	0.045	0.584	0.303	0.052
Congo	0.003	0.021	0.11	0.242	0.532	0.049
India	0.031	0	0.004	0.086	0.117	0.049
Sudan	0.035	0.026	0.122	0.783	0	0.047
DRC	0.013	0.063	0.113	0.088	0.575	0.045
Niger	0.009	0.027	0.173	0.455	0.009	0.043
Pakistan	0.084	0.016	0.1	0.068	0.368	0.042
South Sudan	0	0	0.235	0.176	0.235	0.041
Zimbabwe	0.008	0.169	0.151	0.565	0.088	0.040
Côte d'Ivoire	0.005	0.062	0.282	0.393	0.197	0.040
Lesotho	0.021	0.025	0.348	0.188	0.392	0.039
Cameroon	0.019	0.081	0.28	0.143	0.453	0.039
Cambodia	0.113	0.057	0.234	0.453	0.057	0.037
Papua New Guinea	0	0	0.369	0.184	0.073	0.032
Nigeria	0.052	0.109	0.338	0.23	0.181	0.032
Mozambique	0.027	0.066	0.453	0.244	0.155	0.032
Viet Nam	0.492	0.002	0.029	0.208	0.206	0.032
Togo	0.036	0.204	0.305	0.114	0.323	0.031
South Africa	0.019	0.067	0.473	0.105	0.245	0.030
Gambia	0.046	0.077	0.462	0.231	0.123	0.030
Nepal	0.033	0.078	0.208	0.105	0.099	0.030
Haiti	0.004	0.063	0.538	0.063	0.278	0.030
Myanmar	0.055	0.019	0.537	0.267	0.019	0.029
Guinea-Bissau	0.249	0.245	0.047	0.066	0.284	0.028
Guinea	0.047	0.168	0.177	0.168	0.037	0.027
Indonesia	0.085	0.082	0.506	0.211	0.044	0.026
Liberia	0	0.112	0.607	0.214	0.049	0.026
Sierra Leone	0.007	0.205	0.504	0.279	0.002	0.025
Ghana	0.019	0.282	0.277	0.176	0.083	0.025
Rwanda	0.025	0.169	0.511	0.169	0.079	0.025
Chad	0	0.231	0.462	0.077	0.179	0.024
Zambia	0.015	0.167	0.54	0.16	0.063	0.024
Kenya	0.059	0.182	0.479	0.141	0.079	0.023
Mali	0.015	0.308	0.416	0.231	0.006	0.023
Uganda	0.041	0.173	0.513	0.055	0.114	0.022
Senegal	0.08	0.314	0.378	0.154	0.059	0.021
Madagascar	0.021	0.149	0.622	0.121	0.028	0.021
Burundi	0.041	0.26	0.486	0.068	0.082	0.020

Benin	0.129	0.435	0.177	0.129	0.073	0.020
Burkina Faso	0.046	0.44	0.319	0.127	0.059	0.019
Malawi	0.018	0.199	0.498	0.038	0.058	0.019
Ethiopia	0.022	0.241	0.635	0.072	0.014	0.018
Uzbekistan	0.801	0.002	0.042	0.037	0.035	0.017

^ Of the 70 countries in this analysis 44 has contraceptive method-specific data. The other 26 countries were assigned a simple average of CFRs globally (0.031).

*Calculated as (A*0.014 IUD CFR +B*0.006 implant CFR +C*0.017 injectable CFR +D*0.055 pill CFR +E*0.054 male condom CFR)/ (A+B+C+D+E)
