# Supplementary material for: The role of contraception in preventing HIV-positive births: global estimates and projections
Source: BMC Public Health. 2021 Mar 19;21:536. doi: 10.1186/s12889-021-10570-w (PMC7977320; doi:10.1186/s12889-021-10570-w)
Supplement: Supplementary file 2 — Additional file 2. [file 12889_2021_10570_MOESM2_ESM.pdf]

Additional File 2: Number of HIV positive births averted by contraception annually, 70 countries

Country	WLHIV aged 15-49[&] (A)	WLHIV (15-49) on modern contraception[§] (B)	Pregnancies to WLHIV averted by contraception[¶] (C)	Births to WLHIV averted by contraception [±] (D)	HIV- positive births averted by contraception[#]		
					Treated	Untreated	Total
South Africa	4050000	2187000	204530	127930	4452	4989	9441
Nigeria	860000	161680	36138	22604	398	3798	4195
Kenya	740000	434380	96648	55337	2014	1494	3508
Zimbabwe	600000	394800	81224	46506	1749	837	2586
India	655544	313350	26808	14898	358	1788	2145
Malawi	480000	278880	63411	36307	1380	545	1924
Uganda	650000	235950	52899	30288	1127	636	1763
Indonesia	217800	128066	12919	6709	40	1711	1751
Zambia	580000	259260	57572	32963	1253	494	1747
Mozambique	1060000	268180	57384	32856	1249	493	1741
Tanzania	720000	230400	50511	28921	1076	607	1683
Ethiopia	324000	122472	27981	16021	590	384	974
Cameroon	277000	58170	13972	8748	280	525	805
Angola	179000	22375	5549	3474	53	646	699
Brazil	244313	189831	16895	8172	278	368	646
Ghana	170000	42500	9822	6144	194	387	581
Lesotho	152000	90896	7737	4840	149	334	483
DRC	234000	17550	4105	2570	45	432	477
Mali	74000	11174	2607	1631	16	372	387
Eswatini	103000	67465	6274	3924	124	247	371
Côte d'Ivoire	205000	40180	8682	5431	195	163	358
Thailand	170000	128350	12322	6399	243	96	339
Rwanda	103000	48925	10823	6197	235	93	328
Congo	46900	8677	1999	1252	13	282	294
U.S.	226698	153475	10590	5509	209	83	292
Russia	278415	153128	12097	5068	193	76	269
Botswana	159000	81408	7571	4736	180	71	251
Madagascar	18578	7171	1613	924	4	247	251
Myanmar	85000	43605	4287	2226	71	134	205
Viet Nam	60000	39000	3706	1925	62	110	172
Colombia	21300	16167	1439	696	6	165	171
Pakistan	44700	11175	1030	573	2	155	157
Mexico	26000	16796	1579	866	17	132	149
Namibia	87000	48111	4474	2799	106	42	148
Uzbekistan	13700	8480	990	640	9	125	134
China	89482	72033	4898	2278	87	34	121
Togo	53900	9271	2084	1304	42	78	120

Senegal	20800	5450	1278	800	21	84	105
Venezuela	22233	13718	1646	591	11	92	103
Gabon	29600	5742	1424	892	26	75	101
Haiti	73000	23214	2399	1192	40	61	100
Burkina Faso	42000	12558	2976	1861	71	28	99
Central African Republic	48000	5808	1320	826	23	72	95
Burundi	34300	7683	1738	995	32	60	92
Guinea	57300	4469	1025	641	17	67	84
Guatemala	14900	7286	685	376	5	74	79
South Sudan	87000	4350	893	511	11	67	79
Sierra Leone	34300	7477	1724	1078	38	36	74
Guinea-Bissau	20400	2938	671	419	8	65	73
Dominican Republic	25800	17544	1789	889	30	43	73
Equatorial Guinea	26300	2499	620	388	8	58	66
Chad	52300	2615	666	417	9	55	64
Ukraine	72000	34416	2719	1139	43	17	60
Argentina	30000	23460	2088	1010	38	15	54
Sudan	22700	2656	257	177	0	50	51
France	26000	18902	926	564	19	30	49
Liberia	18500	5550	1277	799	30	17	46
Nepal	10100	4323	450	250	5	37	42
Iran	13200	7524	775	431	14	25	39
Cambodia	25000	9700	872	453	15	20	36
Papua New Guinea	20000	4860	406	365	12	23	35
Benin	34900	4188	990	619	24	9	33
United Kingdom	19030	15985	991	549	21	8	29
Niger	12900	1355	289	181	4	23	27
Peru	12700	6922	616	298	10	13	24
Italy	20000	10360	508	234	8	13	20
Gambia	12300	996	225	141	4	14	17
Ecuador	10000	7170	638	309	12	5	16
Spain	11000	7689	377	174	6	9	15
Malaysia	11700	4013	385	200	8	3	11

& Calculated as women living with HIV aged 15+ - WLHIV aged 50+

§ Calculated as A * Contraceptive prevalence rate

¶ Calculated as (B * pregnancy rate) – (B * contraception failure rate)

± Calculated as C * proportion of pregnancies resulting in live birth (birth rate/pregnancy rate)

Calculated as (D * Coverage of pregnant women for preventing mother-to-child transmission [PMTCT])* 0.04 estimated transmission among those on treatment) + (D * (1 – coverage of pregnant women for PMTCT) * 0.30 estimated transmission among those not on treatment)
