# Supplementary material for: The role of contraception in preventing HIV-positive births: global estimates and projections
Source: BMC Public Health. 2021 Mar 19;21:536. doi: 10.1186/s12889-021-10570-w (PMC7977320; doi:10.1186/s12889-021-10570-w)
Supplement: Supplementary file 3 — Additional file 3. [file 12889_2021_10570_MOESM3_ESM.pdf]

Additional File 3: Number of additional infant HIV infections averted if unintended births to WLHIV were prevented, 70 countries

Country	No of WLHIV aged 15-49[&] (A)	No of births to WLHIV annually[§] (B)	No of unintended births to WLHIV annually[¶] (C)	Total new infant infections from unintended births to WLHIV[#]		
				Treated	Untreated	Total
South Africa	4050000	302024	163093	5676	6361	12036
Nigeria	860000	142123	14923	263	2507	2770
Uganda	650000	105659	43848	1631	921	2552
DRC	234000	43479	12522	220	2104	2324
Angola	179000	31753	10669	162	1984	2147
Tanzania	720000	112564	34895	1298	733	2031
Kenya	740000	82445	29433	1071	795	1866
Zambia	580000	86809	33335	1267	500	1767
Malawi	480000	67712	27694	1052	415	1468
Mozambique	1060000	167026	24720	939	371	1310
Zimbabwe	600000	69895	22716	854	409	1263
Cameroon	277000	40647	9633	308	578	886
Lesotho	152000	15567	8017	247	553	800
South Sudan	87000	12901	4773	107	630	737
Ethiopia	324000	43585	11071	407	266	673
Eswatini	103000	9982	6359	201	401	602
Ghana	170000	20201	6242	197	393	590
Indonesia	217800	14774	2246	13	573	586
India	655544	45359	3946	95	474	568
Brazil	244313	12491	6870	234	309	543
Côte d'Ivoire	205000	30598	7405	267	222	489
Mali	74000	13867	1941	19	443	461
Congo	46900	6392	1796	18	404	422
Botswana	159000	14168	7651	291	115	406
Haiti	73000	6685	3790	126	193	319
Namibia	87000	9485	4847	184	73	257
Rwanda	103000	12931	4629	176	69	245
U.S	226698	13041	4564	173	68	242
CAR	48000	7353	2059	58	179	238
Equatorial Guinea	26300	3984	1116	22	167	190
Togo	53900	7288	2041	65	122	188
Chad	52300	9897	1198	27	158	185
Sudan	22700	3040	638	1	182	183
Pakistan	44700	5097	617	2	167	169
Burundi	34300	5742	1820	58	109	167
Gabon	29600	3728	1454	42	122	164

Guinea	57300	8657	1247	32	131	163
Colombia	21300	1178	594	5	141	146
Venezuela	22233	1508	829	16	129	145
Mexico	26000	1684	775	15	119	134
Russian Federation	278415	14186	1986	75	30	105
Guatemala	14900	1371	488	7	97	103
Guinea-Bissau	20400	2887	491	9	77	86
Madagascar	18578	2493	307	1	82	83
Thailand	170000	7056	1552	59	23	82
Senegal	20800	2931	592	15	62	78
Viet Nam	60000	3703	848	27	48	76
Dominican Republic	25800	1927	919	31	44	75
Argentina	30000	2018	1110	42	17	59
Papua New Guinea	20000	2166	600	19	38	57
Benin	34900	5378	914	35	14	48
Liberia	18500	2561	781	29	16	45
Sierra Leone	34300	4742	640	23	21	44
Myanmar	85000	5396	469	15	28	43
Uzbekistan	13700	1068	192	3	37	40
Peru	12700	874	479	16	22	38
Niger	12900	2852	234	5	29	35
Gambia	12300	1988	260	7	25	32
Burkina Faso	42000	6886	565	21	8	30
Cambodia	25000	2056	319	11	14	25
China	89482	4199	420	16	6	22
Ecuador	10000	756	416	16	6	22
Nepal	10100	667	125	3	18	21
Ukraine	72000	2773	388	15	6	21
France	26000	1356	217	7	12	19
Iran	13200	864	156	5	9	14
Italy	20000	718	136	4	7	12
Malaysia	11700	735	162	6	2	9
Spain	11000	397	75	2	4	7
United Kingdom	19030	986	108	4	2	6

& Calculated as women living with HIV (WLHIV) aged 15+ - WLHIV aged 50+

§ Calculated as A * annual birth rate

¶ Calculated as B * proportion of births that are unintended

Calculated as (C * coverage of pregnant women for PMTCT * 0.04 estimated transmission among those on treatment) + (C * (1 - coverage of pregnant women for PMTCT) * 0.3 estimated transmission among those on treatment)
